# Transmission of LG Modes in High-Capacity 16 × 10 Gbps FSO System Using FBG Sensors Under Different Channel Scenarios

**DOI:** 10.3390/mi16070738

**Published:** 2025-06-24

**Authors:** Meet Kumari, Satyendra K. Mishra

**Affiliations:** 1 Department of ECE, UIE, and UCRD, Chandigarh University, Mohali 140413, Punjab, India; meetkumari08@yahoo.in; 2 SRCOM Division, Centre Technologic de Telecomunicacions de Catalunya, Castelldefels, 08860 Barcelona, Spain

**Keywords:** fiber Bragg grating (FBG), free space optics (FSO), gamma–gamma, Laguerre-Gaussian (LG), log-normal, sensor, turbulence, weather conditions

## Abstract

Free space optics (FSO) aims to perform as one of the best optical wireless channels to design a reliable, flexible, and cost-effective communication system. In FSO systems, mode-division multiplexing (MDM) transmission is a proven technique to expand transmission capacity per communication link. Thus, a 16 × 10 Gbps MDM-FSO system using fiber Bragg grating (FBG) sensors for the coexistence of communication and sensing, exploiting FSO links to transmit distinct Laguerre-Gaussian (LG) beams at a 1000–1900 m range, is proposed. The results illustrate that the system can transmit higher-order LG beams with sensor temperatures of 20–120 °C over a 1500 m range under clear air, drizzle, and moderate haze weather. Also, an improved performance is achieved in gamma–gamma compared to the log-normal distribution model for 10^−6^–10^−2.5^ index modulation under weak-to-strong turbulence. The proposed system is capable of offering a high optical signal-to-noise ratio (OSNR) and gain of 113.39 and 15.43 dB, respectively, at an aggregate data rate of 160 Gbps under different atmospheric scenarios. Moreover, the proposed system achieves better system performance compared to existing works.

## 1. Introduction

A major aspect in the rise of future networks relates to the integration of sensing elements in unified network architecture. Coexisting systems, which influence operational fiber architecture in carriers’ networks as both communication medium and sensing, have already been effectively demonstrated for a collection of sensing applications [1]. Sensors play a crucial role in numerous daily activities of individuals. At present, fiber Bragg grating (FBG) sensors are the most favorably used type of sensors because of their high-multiplexing potentiality, small size, high sensitivity, and immunity to electromagnetic interference, making them important for distinct applications, like aerospace, energy, and defense. In contrast, in the integration of sensor networks and optical communication, problems arise concerning the reliability and flexibility of the network when installing fiber cables to transmit data and sensing signals in long-haul transmission systems. Nevertheless, topographical constraints, like mountains, rivers, buildings, and others, are some difficulties in installing optical fibers. Recently, to address these issues, a flexible, easily configurable, and cost-effective free space optics (FSO)-based scheme has been proposed [2,3].

Free-space optical (FSO) technology has arisen as a prerequisite technology that provides high-speed information transmission without reliance on a physical channel. FSO communications exhibit several benefits over conventional radio frequency (RF) communications, comprising enhanced security, greater bandwidth, and reduced risk of interference. Nevertheless, FSO technology also experiences severe challenges, notably the requirement for precise pointing to sustain the alignment of communication channels over long distances. This need underscores the significance of robust as well as accurate channel models [4]. Moreover, FSO is highly susceptible to atmospheric conditions like haze, fog, dust, rain, etc., which can greatly degrade performance, and its consistency on line-of-sight (LoS) makes it liable to interruptions initiated by obstacles blocking the signal communication path. To address these issues, hybrid FSO and space division multiplexing (SDM) systems have been illustrated, which integrate SDM schemes for FSO links [5].

Further, along with the gradual development of network technology, the digital era is booming forward, the market for high data transmission capacity is escalating, and the conventional fiber-based communication scenario is not far from the capacity limit, having reached an obstruction. SDM technology emerges as an effective solution to break through the present constraints in the future. In the SDM mode division multiplexing (MDM) approach, with few-mode fiber or FSO as the transmission channel, only a single channel can be divided into several modes for the sake of the instantaneous transmission of multiple signals and effectively upgrading data capacity. This simultaneous transmission of data signals makes MDM certainly one of the promising solutions for next-generation networks [6]. In the past few years, the MDM approach has attracted steadily more attention owing to its ability to further enhance the transmission capacity by incorporating high-order modes [7].

Notably, MDM-based FSO systems combine the high security and bandwidth of FSO with the atmospheric resilience, high capacity, and cost-effectiveness of MDM, furnishing a more robust solution for long-haul wireless communication. This system provides an adequate solution for creating flexible, reliable, and cost-effective networks, encouraging optical access as well as sensor network technologies [3]. In the MDM-based FSO system, intensity and modal multiplexing techniques are utilized to enhance the multiplexing capacity of a sensor network by more than twice the traditional FSO technology.

### 1.1. Related Work

In recent years, distinct conventional FSO systems have been presented to determine the sensor’s sensing signal. An integrated intensity and wavelength division multiplexing (IWDM)-based FBG sensor system over a 2 m FSO transmission channel is proposed. This system used the stacked gated recurrent unit (SGRU) algorithm along with a neural network (NN) profile scheme at a limited data rate of 2.5 Gbps [8]. Also, a bidirectional IWDM-based FBG sensor network using a coarse wavelength division multiplexing (CWDM) approach over a 25 km single-mode fiber (SMF) and 2 m FSO range is presented. The results illustrate that the system employing hybrid stacked gated recurrent units and long short-term memory (SGRU-LSTM) model offers a long-distance strain-sensing facility. Accordingly, several parameters, including hidden layers, epochs, batch sizes, activation functions, and optimizers are adjusted to measure the optimal values [3]. Likewise, an integrated FSO/RF system over a 2 km FSO range under the impact of air, rain, and snow conditions is simulated. However, the utilization of the complex Malaga channel model makes system performance analysis complex and less reliable [5]. An FSO system using a photonic sensor at a 10 Gbps data rate is realized. Also, the results depict that the system employing polarization multiplexing can provide an integrated 6 km SMF and 100 m outdoor FSO channel with <2% discrepancy for all temperature measurements [1]. Further, some of the latest works use the MDM scheme in FSO systems when considering different atmospheric conditions. A 120 Gbps integrated orthogonal frequency division multiplexing (OFDM)-based MDM system using orbital angular momentum (OAM) modes is transmitted over a 1.5 and 0.4 km FSO range under low and heavy dust, respectively. Even with a high input power of 20 dB, this system offers a bounded optical-signal-to-noise ratio (OSNR) of 30 dB under strong turbulence [9]. Like this, an MDM-FSO system using OAM modes is realized at a 2 Gbps data rate under weak and strong turbulence [10].

From the detailed prior study, it is understandable that integrated FSO systems with the MDM approach, with or without employing sensors, are still limited in long-reach high-speed transmission under different atmospheric conditions.

### 1.2. Motivation

The growing demand for high internet traffic, which is an existing attribute of the increasing prominence of 5G services, cloud computing, big data, etc., keeps growing in the world. To compete with this challenge, high-capacity MDM-FSO systems have been widely developed, where high-capacity transceivers can combat atmospheric turbulence [11]. Further, one of the most widely used and highly versatile sensors, i.e., FBG, is utilized to monitor and track various physical parameters like slope, pressure, acceleration, strain, temperature, displacement, and load. The emergence of FSO technology provided a solution to optical fiber geographical constraints, like rivers, mountains, etc., enabling the optical sensing signal transmission. Also, MDM-based FSO technology using sensors is a favorable approach that uses light to transmit information through the free space. The proposed high-capacity MDM-FSO system using FBG sensors can hybrid transmit sensor and Laguerre–Gaussian (LG) beam data, enhancing the reliability and sustainability of the MDM-FSO system under severe atmospheric conditions. This system can transfer sensing information from building to building, solve geographical constraints, and monitor the building. It provides numerous benefits, including high transmission speeds, cost-effectiveness, resistance to harsh areas, high multiplexing ability, secure spectrum licenses, ease of deployment, and low power consumption [3].

### 1.3. Major Contributions

The major contributions of this work are given as follows:

1. To design a high-speed 16 × 10 Gbps MDM-FSO system using uniform FBG sensors.
2. To evaluate the system performance as well as comparison analysis for different LG modes under the impact of different weather conditions, weak-to-strong turbulence, and distribution models, i.e., gamma–gamma (GG) and log-normal (LN).
3. System performance analysis for varied FSO range, variable parameters of sensor (temperature and index modulation), system gain and OSNR, in terms of bit-error-rate (BER) performance, eye patterns, and optical spectra.
4. To validate the feasibility of the proposed sensor-based MDM-FSO system via comparisons with prior works in terms of different parameters.

The remainder of this work is organized as follows. Section 2 describes the system design, detailing the architecture, concept, and design parameters for the proposed system. Section 3 discusses the channel models, obtained results, performance analysis, and validation through comparison analysis. Finally, Section 4 concludes the work along with future research directions.

## 2. Proposed Design

**Figure 1:**
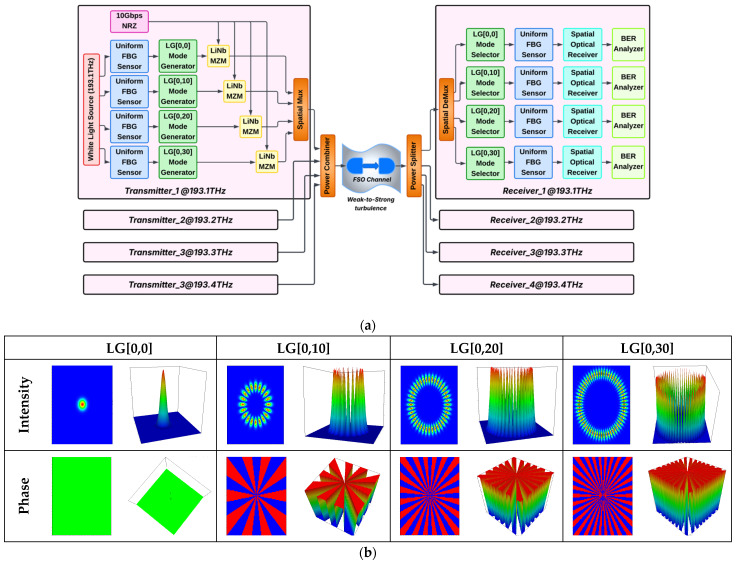
Schematic diagram of (**a**) proposed high-capacity FBG sensor-based FSO system using higher-order LG modes and (**b**) generated LG modes’ profile.

Figure 1a presents the proposed FBG sensor-based MDM-FSO system using different LG modes under different atmospheric conditions. Figure 1b illustrates the generated mode profiles in both 2D and 3D views. This system is designed and investigated in OptiSystem v.21.

In the proposed FSO system, the purpose of using FBG sensors at both Tx and Rx sides is to localize atmospheric sensing (temperature, strain, pressure, vibration) as well as FSO link monitoring. The FBG sensors used in different environments (at Tx/Rx) operate independently to monitor different physical parameters and thus reflect unique wavelength shifts based on the local atmospheric conditions. It also improves channel estimation, detects redundancy, and diagnoses faults in the proposed system.

This system setup includes three key components, viz., FBG sensor, LG mode generator, and FSO link. The transmitter section consists of four sub-transmitters operating at different terahertz frequencies at 193.1, 193.2, 193.3, and 193.4 THz. A white light source operating at a specific frequency with an input power of 10 dBm and a 10 MHz linewidth is used to transmit the optical signal. Four uniform FBG sensors are used, where each sensor is an optical fiber sensor based on the principles of Bragg reflection. The sensing optical signal is passed to a specific LG mode generator to transmit four different LG beams, viz., LG[0,0], LG[0,10], LG[0,20], and LG[0,30]. After this, the generated sensor-based LG beams are modulated with non-return-to-data signals at a 10 Gbps data rate/channel via a LiNb Mach-Zehnder modulator (MZM). A spatial multiplexer multiplexes all LG beam signals operating at a specific frequency, and then all combined beams from all transmitters, offering an aggregate data rate of 16 × 10 Gbps, are passed through an FSO channel. An FSO channel is realized under the impact of clear weather, haze, weak-to-strong turbulence, and geometric loss. At the receiver, a power splitter distributes power to different receive sub-sections, where incoming integrated sensing and LG beams are de-multiplexed to specific LG mode selectors followed by a uniform FBG sensor. For each incoming beam, a spatial optical receiver generates an electrical signal along with providing a low-noise signal by using a low-pass filter. BER performance and eye patterns are achieved using a BER analyzer. Generally, LG modes are represented as [12]:

(1) $$\Phi_{s,l}\left(u,ø\right)=\alpha {\left(\frac{2u^{2}}{\omega_{0}^{2}}\right)}^{\frac{\theta }{2}}.L_{s}^{l}\left(\frac{2u^{2}}{\omega_{0}^{2}}\right).exp\left(\frac{-r^{2}}{\omega_{0}^{2}}\right).exp\left(\frac{\pi u^{2}}{\lambda R_{0}}\right)\left\{\begin{array}{c} \cos\left(lø\right),\;l<0\\ \sin\left(lø\right),\;l\geq 0 \end{array}\right.$$

where u, $\omega_{0}$, λ and $R_{0}$ mean curvature radius, spot size, wavelength, and normalized radius, respectively. s and l stand for mode dependencies in the x-and y-axes, respectively, $L_{s}^{l}$ indicates Laguerre polynomial, θ and α stand for beam divergence and atmospheric attenuation coefficients, respectively. Table 1 illustrates various simulation parameters used in the system.

At the transmitter and receiver side, a 4-way power combiner/splitter comprises an insertion loss of ~2 dB with 0.5 dB loss per path. Four FBG sensors per Tx/Rx section also introduce loss (~2 dB) depending on their design, and the generated higher-order modes from the mode generator increase insertion loss (~2 dB) owing to coupling mismatches. Thus, cumulative insertion loss per path exceeds ~20 dB in the proposed system. High insertion loss reduces the received power, deteriorating SNR and thus high BER. For this, lower-order mode generators and array waveguide generator (AWG) multiplexer/de-multiplexer can be used with a tradeoff between system gain and link margin.

## 3. Results and Discussions

In the work, both the gamma–gamma and log-normal channel models under weak-to-strong turbulence as well as a variety of weather conditions are introduced to evaluate the performance of the system. Also, the geometric loss, additional loss, transceiver loss, and free space path loss are included.

### 3.1. System Performace Under Atmospheric Conditions w.r.t. FSO Link

In this subsection, the system performance is analyzed for varied FSO ranges under the impact of gamma–gamma medium turbulence and different weather conditions for all LG modes.

In FSO networks, terrestrial communication links between mountain-to-mountain, building-to-building, or any other type of horizontal link between two ground stations experience power loss owing to several factors. When an optical beam propagates through the atmosphere, it undergoes absorption loss, scattering loss, atmospheric turbulence, beam divergence loss, ambient light, and misalignment. Also, FSO communication, including ground-to-satellite and vice-versa links, experiences pointing loss, atmospheric turbulence-vertical, background noise, atmospheric seeing, and angle of arrival fluctuations [14]. Mostly, in urban or industrial regions, drizzle-moderate haze (visibility ~1–3 km, with aerosol concentration of ~100–300 µg/m^3^) can occur usually up to 20–40% of days in winter. In cleaner areas, the probability is <5–10% annually in India. Thus, the geographical location of FSO networks is very critical, as the climatic conditions and the wind determine the link availability of the system.

Sources, such as smoke, dust, and other particles, which are distributed in the atmosphere, induce particulate matter known as haze. Exposure of these particles to gaseous pollutants causes certain chemical reactions [15]. Haze atmospheric attenuation is dependent on wavelengths between 785 and 1550 nm. Generally, haze particles are 0.01–1 µm size; thus, optical beams suffer from less attenuation as compared to fog, snow, and rain conditions [16]. Also, the wavelength 1550 nm is especially suitable as it can cover long distances, deal with high transmission rates, and reduce the solar background, light scattering, and absorption in case of haze [17]. Haze causes Rayleigh/Mie scattering if particle size is smaller/comparable with the operating wavelength.

To mitigate the impact of different weather conditions on FSO systems, adequate aperture averaging, modulation speed, and specific wavelength are highly important. The aperture averaging technique is utilized by increasing the receiver aperture size, which averages out fast enough fluctuations generated by small-size eddies and helps in minimizing channel fading. It is identified by aperture averaging factor, A, as [14]:

(2) $$A=\frac{\sigma_{I}^{2}\left(D_{r}\right)}{\sigma_{I}^{2}\left(0\right)}$$

where $\sigma_{I}$ is the variance of the signal fluctuations at the receiver with a receiver aperture diameter of $D_{r}$. As the optical signal propagates through a hazy atmosphere, a beam divergence is generated by diffraction at the receiver. Due to this, some fractions of the incoming beam will not be acquired by the receiver, which will cause geometrical/beam divergence loss. This loss increases with the FSO link distance unless the receiver aperture size is increased or receiver diversity is incorporated [14]. Moreover, the on–off keying (OOK) modulation technique is mostly used in FSO systems with an intensity modulated/direct-detection (IM/DD) receiver mechanism owing to its simplicity. The combination of OOK modulation and adequate receiver aperture size at 1550 nm wavelength has proven the feasibility of enhanced FSO systems through high-speed, low-complexity, and cost-effective designs. Meanwhile, for optical-to-electrical semiconductor receivers, the beams are focused on a high-quality spatial germanium or indium gallium arsenide (InGaAs) PIN or avalanche photodetector (PD) [10]. InGaAs PIN PD shows good response to low attenuation window wavelengths, i.e., 1550 nm, while InGaAs APD PD has a higher output current compared to PIN PD at specific input power. However, in APD PD, the noise also increases by the same factor as well as additionally, and thus it offers a slower response than PIN PD.

Simulation BER results are presented along with eye patterns, and the results are compared with prior approaches.

The probability density function (PDF) of the gamma–gamma model is expressed as [18]:

(3) $$f_{h_{g}}\left(h_{g}\right)=\frac{2{\left(m_{g}n_{g}\right)}^{\left(\left(m_{g}+n_{g}\right)/2\right)}}{\Gamma \left(m_{g}\right)\Gamma \left(n_{g}\right)}.{h_{g}}^{\left(\left(m_{g}+n_{g}\right)/2\right)-1}K_{m_{g}-n_{g}}\left[2\sqrt{\left(m_{g}n_{g}h_{g}\right)}\right]$$

where $h_{g}$ stands for fading coefficient, $\Gamma \left(.\right)$ and $K_{m_{g}-n_{g}}\left(.\right)$ stand for gamma function and second-kind modified Bessel function of order $\left(m_{g}-n_{g}\right)$, respectively, $m_{g}$ and $n_{g}$ stands for the effective number of large- and small-scale eddies. These are expressed as [18]:

(4) $$m_{g}={\left[exp\left\{\frac{0.49k_{0}^{2}}{{\left(1+1.11k_{0}^{\frac{12}{5}}\right)}^{\frac{7}{6}}}\right\}-1\right]}^{-1}$$

and

(5) $$n_{g}={\left[exp\left\{\frac{0.51k_{0}^{2}}{{\left(1+0.69k_{0}^{\frac{12}{5}}\right)}^{\frac{5}{6}}}\right\}-1\right]}^{-1}$$

where $k_{0}^{2}$ stands for the scintillation index.

Furthermore, in FSO, channel gain is given as [19]:

(6) $$h=h_{l}h_{p}h_{a}$$

where $h_{p}$, $h_{l}$ and $h_{a}$ stand for channel fading owing to path loss, geometric spread, and turbulence, respectively. The received power, $P_{r}$, at receiver is given as [14]:

(7) $$P_{r}=P_{t}.\;\left(\frac{A_{r}}{{\left(\theta L\right)}^{2}}\right).exp\left(-\alpha L\right)$$

where $P_{t}$ stands for power transmitted, $A_{r}$ stands for effective receiver aperture area. Again, to calculate haze attenuation, the Beer–Lambert Law is given as [14]:

(8) $$\alpha_{haze}=e^{-\sigma L}$$

where σ stands for the haze attenuation coefficient and is given as $\frac{3.91}{V}{\left(\frac{\lambda }{550}\right)}^{-p}$. The coefficient, p (in Kruse and Kim’s model) with visibility, V can be expressed as [14]:

(9) $$p=\left\{\begin{array}{l} 1.6\;\;\;\;\;\;\;\;\;\;\;\;\;\;\;\;\;\;\;for\;V>50\;\mathrm{km}\\ 1.3\;\;\;\;\;\;\;\;\;\;for\;6<V<50\;\mathrm{km}\;\;\;\;\;\;\;\;\;\;\;\;\left[\mathrm{Kruse}\;\mathrm{model}\right]\\ 0.585V^{1/3}\;\;\;\;\;\;\;\;\;\;for\;V<6\;\mathrm{km} \end{array}\right.$$

and

(10) $$p=\left\{\begin{array}{l} 1.6\;\;\;\;\;\;\;\;\;\;\;\;\;\;\;\;\;\;\;\;\;\;\;\;\;\;\;\;\;\;\;\;\;\;\;\;\;\;\;for\;V>50\;\mathrm{km}\\ 1.3\;\;\;\;\;\;\;\;\;\;\;\;\;\;\;\;\;\;\;\;\;\;\;\;\;\;\;\;\;\;\;\;for\;6<V<50\;\mathrm{km}\\ 0.16V+1.344\;\;\;\;\;\;\;\;\;\;\;\;\;\;\;for\;1<V<6\;\mathrm{km}\\ V-0.5\;\;\;\;\;\;\;\;\;\;\;\;\;\;\;\;\;\;\;\;\;\;\;for\;0.5<V<1\;\mathrm{km}\\ 0\;\;\;\;\;\;\;\;\;\;\;\;\;\;\;\;\;\;\;\;\;\;\;\;\;\;\;\;\;\;\;\;\;\;\;\;\;\;\;\;\;for\;V<0.5\;\mathrm{km} \end{array}\right.\;\;\left[\mathrm{Kim}’s\;\mathrm{model}\right]$$

The BER performance can be expressed as [14]:

(11) $$BER=1/2erfc\sqrt{SNR/8}$$

where erfc is the complementary error function and SNR stands for the signal-to-noise ratio.

In the proposed system, higher-order LG modes of 0, 10, 20, and 30 are used to evaluate the system performance. Higher-order modes comprise more orthogonality, allow more independent channels, scale the system capacity with the given bandwidth, lower inter-modal interference, and enhance spatial diversity. Due to all these reasons, the system comprising higher-order LG modes can be used for ultra-dense MDM systems with enhanced spectral efficiency and data throughput.

**Figure 2:**
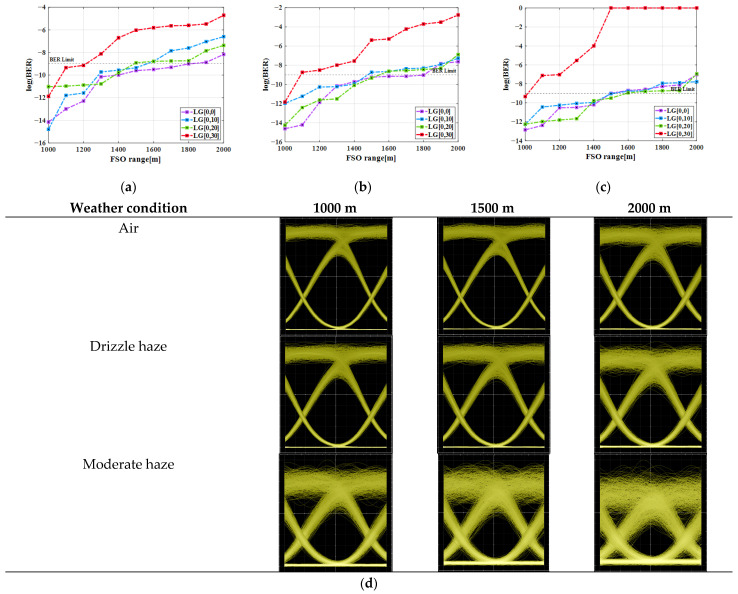
BER vs. FSO range for different LG modes under (**a**) clear air, (**b**) drizzle haze, (**c**) moderate haze, and (**d**) corresponding eye patterns at distinct FSO range for LG[0,0] mode.

Figure 2a–c shows simulated BER values over a 1000–2000 m FSO range under clear air, drizzle, and moderate haze, respectively, at an aggregate data rate of 160 Gbps. As illustrated, the maximum FSO transmission ranges under clear air, drizzle, and moderate haze at a BER limit of 10^−9^ are ~1900, 1800, and 1550 m, respectively, for LG[0,0] mode. However, the communication range decreases for higher-order modes, viz., LG[0,10] followed by LG[0,20] and LG[0,30]. This is due to the reason that higher-order modes have greater beam divergence, more misalignment sensitivity, lower coupling efficiency, high sensitivity to turbulence and scattering, and lower power concentrations. Thus, FSO ranges of 1600, 1510, and 1500 m for LG[0,10]; 1550, 1500, and 1500 m for LG[0,20]; and 1200, 1100, and 1000 m for LG[0,30] are obtained under air, drizzle, and moderate haze scenarios, respectively. In the proposed system, LG[0,30] mode shows much worse BER performance than the other three due to greater beam divergence, higher energy loss, and greater sensitivity to atmospheric conditions (drizzle/moderate haze over weak-to-strong turbulence). Also, the increased modal distortion, channel crosstalk, and detection challenges for LG[0,30] mode lead to a low system performance in realistic channel conditions.

Further, Figure 2d illustrates the generated eye patterns under different weather conditions at 1000, 1500, and 2000 m for fundamental mode {LG[0,0])}. The lithium niobate (LiNbO_3_) MZM modulator plays a major role in determining the rise time, fall time, and 0/1 level broadening in the eye diagram. Besides the MZM’s bandwidth and extinction ratio, the final eye shape is affected cumulatively by FSO link impairments (e.g., Rayleigh and Mie scattering, absorption, attenuation, and turbulence), pulse shaping, transmission rates, and optical receiver characteristics. These clear eye patterns mean that transmitted LG beams are of high quality without noticeable degradation compared to other weather conditions. Atmospheric absorption due to aerosols, molecules, or absorption is less than 0.01 dB/km in clear air at 1550 nm. However, the impact of absorption is quite prominent in drizzle haze followed by moderate haze. Also, if the size of atmospheric particles is small and comparable in comparison with the optical wavelength, then Rayleigh and Mie scattering are induced, respectively. Particles like air molecules, aerosols, and haze are major contributors to Rayleigh and Mie scattering. In addition, atmospheric turbulence leads to the loss of spatial coherence of incoming coherent beams, beam spreading, depolarization, and temporal stretching of the light pulse [14]. Due to all these factors, the clear and widely opened eye patterns are seen under clear air compared to drizzle haze followed by moderate haze. Meanwhile, the distorted and closed eye patterns obtained in the haze scenario are mainly due to the particle absorption and scattering process in free space. The increase in FSO range further decreases the signal quality majorly in moderate haze owing to high atmospheric attenuation (=4.2 dB/km).

Furthermore, the proposed system achieves better BER performance than existing works in ref. [1,8,9] and ensures a stable and smooth data transmission, exhibiting its adaptability and effectiveness in different weather conditions. Table 2 shows the summarized results achieved with faithful FSO ranges at BER limits for different LG modes under distinct weather conditions.

### 3.2. System Performace Under Atmospheric Conditions w.r.t. Sensor Temperature

In this subsection, the system performance is investigated for varied temperature in the sensor under the impact of gamma–gamma medium turbulence and different weather scenarios for different LG modes. Simulation BER values are presented, and eye patterns are explored.

**Figure 3:**
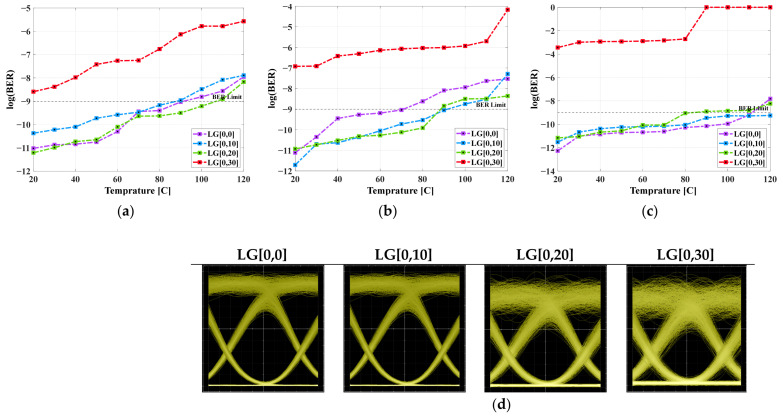
BER vs. sensor temperature for different LG modes under (**a**) clear air, (**b**) drizzle haze, (**c**) moderate haze, and (**d**) corresponding eye patterns at 50 °C for different LG modes under clear weather at 1500 m range.

Figure 3a–c illustrates the BER performance for varied sensor temperatures (20–120 °C) over a 1500 m range at a 160 Gbps data rate under clear air, drizzle, and moderate haze, respectively. It is seen that the BER performance of the system under moderate haze conditions is higher than other conditions, where visibility is largely reduced. Also, in low-visibility drizzle haze conditions, the weather-based system presents better signal quality under clear air. Moreover, the transmitted LG[0,0], LG[0,10], and LG[0,20] beams under different weather conditions gradually increase the BER values compared to the LG[0,30] beam. This is due to higher beam quality, lower divergence, and robustness against atmospheric conditions in lower-order modes. In addition, FBG sensors used in the system offer the unique benefits of monitoring and tracking physical parameters like temperature for a wide range of LG modes under severe weather and turbulence conditions. Meanwhile, it adapts to the existing atmospheric and system conditions, enabling dynamic power allocations to different modes. At a BER of 10−9, maximum temperatures of 20–100 °C under clear air, <20–90 °C under drizzle haze, and <20–120 °C under moderate haze are observed for all LG modes. Here, if the FBG sensor shifts temperature, the LG mode quality degrades. In contrast to ref. [4], in this work, high-quality signals can be obtained up to a maximum temperature of 110 °C.

Figure 3d shows the eye patterns at 50 °C sensor temperature for different LG modes under clear weather at 1500 m range. It is clear that the higher-order LG modes are greatly degraded by the temperature effect, and the BER cannot be evaluated. It is also realized that under clear atmospheric conditions, air particles are negligible, and thus system performance is affected by free space channel path loss as well as system noise. The major distortion in the eye patterns can be attributed to the effect of the higher-order LG mode (LG[0,30]) transmitted at high modulation speeds. After this mode, limited system performance is seen due to lower beam quality and higher beam divergence. Table 3 illustrates the summarized results attained with acceptable BER supporting the sensor’s temperature w.r.t. reference temperature of 0 °C at 1500 m range for different LG modes and distinct weather conditions.

**Figure 4:**
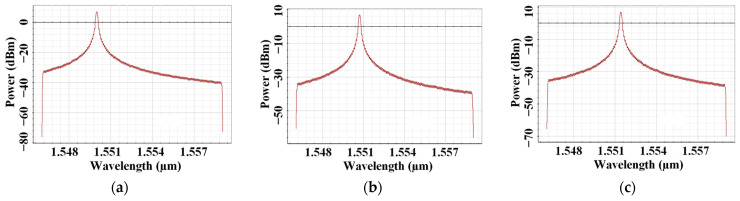
Impact on operating wavelength at (**a**) 20 °C, (**b**) 60 °C and (**c**) 120 °C FBG sensor temperature at 1500 m range.

Figure 4a–c illustrates the shift in wavelength as temperature changes in the FBG sensor at a 1500 m range under clear air for LG[0,0] mode. It is clear that with the rise in temperature, the Bragg wavelength shifts towards a longer wavelength, causing expansion, while in the vice-versa case, contraction can be seen in the figure. As per Bragg wavelength, $\lambda_{Bragg}=2n_{eff}\Delta$, where $n_{eff}$ is the effective refractive index and Δ is the grating period; the design of the FBG affects the optimum operating temperature. To reflect at $\lambda_{Bragg}$ = 1550 nm, the required grading period is ~535 nm, and at 1550 nm, the FBG design will have a thermal sensitivity of ~10.7 pm/°C. In FSO-based systems, this drift can cause signal fading, detection errors, spectral overlap, and high BER. Also, longer gratings provide wider as well as more stable reflection spectra besides tolerating more temperature variation without major misalignment errors [20].

Further, in the proposed system using FBG sensors operating at a specific wavelength, a shift of the power spectrum with different sensor temperatures causes misalignment between transmitter and receiver. This results in lower received optical power, reducing the SNR and thus increasing BER, as seen in Figure 3. Moreover, a temperature-induced Bragg wavelength shift causes channel crosstalk, which degrades the system performance. Also, in FBG-sensing systems, a shifted spectrum is misinterpreted due to the impact of atmospheric conditions.

### 3.3. System Performace Under Different Channel Modes with Atmospheric Turbulence w.r.t. Index Modulation in Sensor

In this subsection, the system performance is analyzed for varied index modulation in FBG sensors under the impact of both gamma–gamma and log-normal channel models. The system performance is analyzed under weak-to-strong turbulence and clear weather for different LG modes in terms of BER values.

Also, the PDF for the log-normal distribution model is defined as [21]:

(12) $$p_{I}\left(I\right)=\frac{exp\left(-\frac{{\left(\ln I-\mu_{y}\right)}^{2}}{2\sigma_{y}^{2}}\right)}{2I\sqrt{2\pi \sigma_{y}^{2}}}$$

where I is instantaneous received optical intensity, $\mu_{y}\;$ is log-amplitude, and $\sigma_{y}^{2}$ is log-intensity variance.

Further, an index of refraction structure $C_{n}^{2\;}$(m^−2/3^) is used to measure the turbulence strength as below [22]:

(13) $$C_{n}^{2\;}={\left(79\times 10^{-6}\frac{P}{T^{2}}\right)}^{2}C_{T}^{2\;}$$

where P, T, and $C_{T}^{2\;}$ stand for atmospheric pressure, average temperature, and temperature structure parameter, respectively. Rytov variance, $k_{0}^{2}$, is given as [5]:

(14) $$k_{0}^{2}=\frac{1}{2}C_{n}^{2\;}{\left(\frac{2\pi }{\lambda }\right)}^{\frac{7}{6}}Z^{\frac{11}{6}}$$

where $k=\frac{2\pi }{\lambda }$ stands for optical wave number. For weak ($C_{n}^{2\;}=10^{-17}{\;m}^{-2/3})$, medium $\left(C_{n}^{2\;}=10^{-15}{\;m}^{-2/3}\right)$, and strong $\left(C_{n}^{2\;}=10^{-13}{\;m}^{-2/3}\right)$ turbulences, $k_{0}^{2}$ values are given as <1, =1 and >1, respectively. Besides this, atmospheric fading strength, SI, is given as [23]:

(15) $$SI=\frac{1}{m_{g}}+\frac{1}{n_{g}}+\frac{1}{m_{g}n_{g}}$$

Further, turbulence strength is defined as the Strehl ratio (SR), which lies between 0 and 1 as [24]:

(16) $$SR={\left[1+{\left(\frac{D_{o}}{r_{o}}\right)}^{5/3}\right]}^{-1}$$

where $D_{o}$ stands for optical beam diameter and $r_{o}=0.185{\left(\frac{\lambda^{2}}{C_{n}^{2}L}\right)}^{5/3}$ means Fried’s parameter, where L is the FSO range. Also, for the proposed work, these parameters’ values are presented in Table 4.

**Figure 5:**
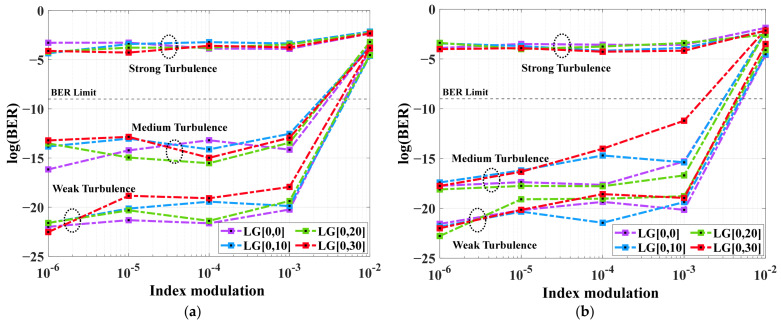
BER vs. index modulation for different LG modes under (**a**) gamma–gamma and (**b**) log-normal FSO models at 1000 m range under air condition.

Figure 5a,b illustrates the BER performance for varied Bragg index modulation values under gamma–gamma and log-normal weak-to-strong turbulent models, respectively, at a 1000 m range for 16 × 10 Gbps different LG beams. It is found that the magnitudes of turbulence effects are strongly dependent on index modulation of the FBG sensor. The proposed system using different LG modes is more sensitive to index modulation, especially under strong turbulence followed by medium and weak turbulence. When index modulation is <10^−2.5^, acceptable BER values are achieved for both weak and medium turbulences for both gamma–gamma and log-normal models. Also, when the modulation index is >10^−2.5^, BER values for both turbulences increase dramatically with the rise in index modulation. On the other hand, the system under strong turbulence undergoes huge signal loss with the increase in index modulation. Obviously, gamma–gamma is better than log-normal, as it reliably models irradiance fluctuations under all turbulence scenarios. The log-normal model is only valid under weak-turbulence conditions, while the gamma–gamma model allows both small-scale and large-scale effects, thus making it accurate for all turbulence scenarios [25]. For 10^−6^ to 10^−2^ index modulation, the BER values are seen as −21.99 to −3.85 and −21.56 to −3.49 under weak turbulence; −16.16 to −3.78 and −19.74 to −4.04 under medium turbulence; and −3.87 to −2.33 and −3.9 to −2.15 under strong turbulence for gamma–gamma and log-normal distribution models, respectively. In addition, system BER, outage probability, and capacity calculations utilizing gamma–gamma offer more reliability in real-world deployment conditions. Also, gamma–gamma distribution fits well measured intensity fluctuations over an extensive range of turbulence. Table 5 illustrates the summarized results obtained in terms of BER for varied index modulation for both distribution models under weak-to-strong turbulence regimes.

### 3.4. Impact of Different Atmospheric Conditions on the System Performance

In this subsection, the system gain and OSNR for 193.1–193.4 THz operating frequencies under different atmospheric conditions and distinct distribution models are presented. Here, the performance is analyzed under medium turbulence and clear weather at a 1500 m range.

**Figure 6:**
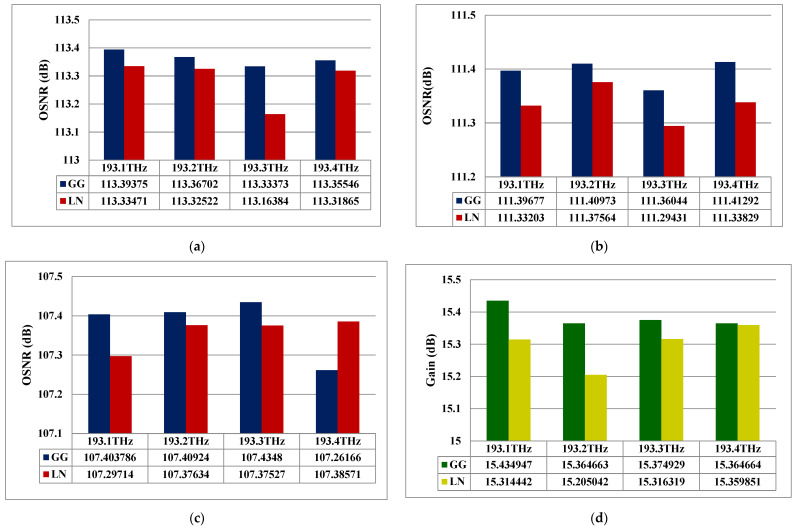

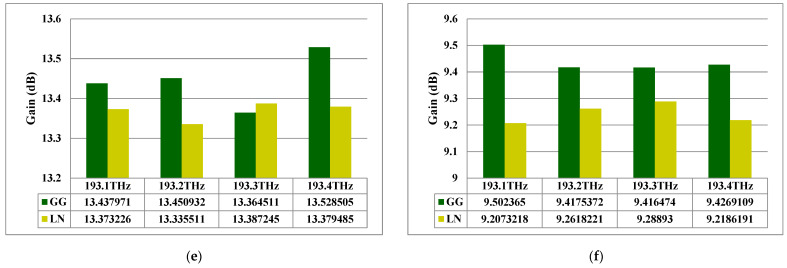
System performance in terms of OSNR under (**a**) air, (**b**) drizzle haze, (**c**) moderate haze; gain under (**d**) air, (**e**) drizzle haze, and (**f**) moderate haze for both gamma–gamma and log-normal models at 1500 m range.

Figure 6a–c exhibits the impact of different weather conditions and distribution models on distinct THz frequencies in terms of OSNR. Clearly, the system using different frequencies offers maximum OSNR as approx. 113.39–113.35 dB under clear weather, 111.39–111.41 dB under drizzle haze, and 107.40–107.26 dB under moderate haze conditions for the gamma–gamma model. While, for the log-normal model, the maximum OSNR is 113.33–113.31, 111.33–111.33, and 107.29–107.38 dB under clear air, drizzle, and moderate haze weather, respectively. Moreover, the system offers maximum gains of 15.43–15.36, 13.43–13.52, and 9.50–9.42 dB under clear air, drizzle, and moderate haze weather conditions, respectively, for the gamma–gamma distribution model, as shown in Figure 6d–f. Also, for the log-normal model with clear air, drizzle, and moderate haze weather, it provides maximum gains of 15.31–15.35, 13.37–13.37, and 9.20–9.21 dB, respectively. It is worth mentioning here that the system performance with the gamma–gamma model is well improved owing to the real-world FSO channel measurement. Noteworthy, the proposed FSO link also affects the signal quality owing to scattering and absorption. In this case, the four different optical tones experience different levels of scattering and absorption loss in addition to attenuation, resulting in an OSNR and gain difference between them. This is especially true for high-speed communication under hazy channel conditions [26].

Mathematically, OSNR and the system gain are defined as [20]:

(17) $$OSNR\;\left(\mathrm{dB}\right)=Signal\;Power\;\left(\mathrm{dB}\right)-Noise\;Power\;\left(\mathrm{dB}\right)$$

and

(18) $$Gain\;\left(\mathrm{dB}\right)=Output\;Power\;\left(\mathrm{dB}\right)-Input\;Power\;\left(\mathrm{dB}\right)$$

### 3.5. System Performance Comparisons with Existing Ones

Table 6 depicts the comparative system performance with prior works in terms of different parameters.

As compared to [1,8,9,10,26], the system offers the highest throughput of 160 Gbps (16 × 10 Gbps) by using quad LG modes under different atmospheric conditions. Also, the system offers reliable and cost-effective signal transmission over a faithful 1900 m range. As compared to existing works in refs. [9,10], where channel capacity is limited to 120 Gbps and 8 × 2 Gbps, respectively, the proposed system offers improvements owing to better turbulence mitigation, a simple modulation scheme (OOK), and adequate input power (=10 dBm). Table 7 illustrates the comparison analysis of the proposed system w.r.t. prior works based on system reliability and cost-effectiveness considering channel and receiver losses. Complex modulation schemes like OFDM and QPSK comprise hardware complexity and less power efficiency and thus offer low-cost IoT-based systems.

The proposed work offers several advantages as compared to existing works, such as high spectral efficiency by using the MDM scheme supporting an aggregate data rate of 160 Gbps, FBG sensors enabling simultaneous coexistence of communication and sensing, and high-bandwidth wireless communication in different atmospheric windows. Thus, this system can be utilized for smart infrastructure, military, and aerospace applications.

Meanwhile, due to the presence of high insertion loss (splitter/combiner, mode generator, and FBG sensors), amplification and power-efficient lasers may be required, therefore increasing system complexity. In the presence of sensitive atmospheric conditions and turbulence, higher-order modes (lg modes) are more susceptible to distortion than lower-order modes. Also, LG modes suffer from mode coupling, reducing the orthogonality and effectiveness of mode multiplexing. On the other hand, temperature-sensitive FBG sensors introduce wavelength signal filtering and distortions.

## 4. Conclusions

A high-speed FBG sensor-based MDM-FSO system using LG beams under different weather conditions, turbulence effects, channel models, and geometric loss is presented. Four higher-order LG beams at [0], [0,10], [0,20], and [0,30] mode index are transmitted along with sensing information. It is concluded that the high-capacity system operating at an aggregate transmission of 160 Gbps exhibits maximum 1900, 1800, and 1000 m transmission ranges under air, drizzle, and moderate haze, respectively. For the same atmospheric conditions, the system offers faithful transmission even in the presence of the highest sensor temperatures of 110, 120, 110, and 20 °C for LG[0,0], LG[0,10], LG[0,20], and LG[0,30] modes, respectively, at a BER of <10−9. In addition, the sensor operating at an index modulation of 10^−6^–10^−2.5^ allows for more successful LG beam transmission for the gamma–gamma model than for the log-normal model, considering weak-to-strong turbulence. It is also concluded that the gamma–gamma FSO model under different weather conditions improves OSNR and gain as 107.26–113.39 and 9.42–15.43 dB, respectively. As compared to prior works, this work is of great significance for facilitating long-distance signal transmission in MDM-FSO-based systems.

In short, this work contributes to promoting FSM-MDM systems employing FBG sensor systems under different FSO link impairments. In the future, this work can be extended for long reach, high capacity, and high speed by integrating an FSO-fiber transmission link for several IoT-based applications. Also, an advanced model can be realized by incorporating deep-learning techniques, system survivability, multiplexing capacity, enhanced transmission distance, measurement accuracy, and cost-effectiveness.

## Figures and Tables

**Table 1:** Simulation specifications [5,13].

Component	Parameters	Value	Unit
Laser	Frequency	193.1–193.4	THz
	Channel spacing	100	GHz
	Input power	10	dBm
Bit generator	Bit rate	10	Gbps
Uniform FBG sensor	Bragg wavelength	1550	nm
	Index modulation	10^−6^ to 10^−2^	
	Length	10	mm
	Temperature	20–120	°C
	Thermal expansion coefficient	5.50 × 10^−7^	/C
	Thermo-optic coefficient	8.60 × 10^−6^	/C
Mode generator	LG[0,0], LG[0,10], LG[0,20], LG[0,30]		
MZM modulator	Extinction ratio	30	dB
FSO	Range	1000–2000	m
	Index refraction structure	10^−13^–10^−17^	m^−2/3^
	Weather condition	0.22 (clear air), 1.5 (Drizzle haze) 4.2 (Moderate haze)	dB/km
	Tx/Rx aperture diameter	10/20	cm
	Transceiver loss	0.1	dB
	Geometric loss	Yes	
	Additional loss	0.1	dB
	Beam divergence	2	mrad
	Free space path loss	Yes	
Spatial receiver	Responsitivity	0.9	A/W
	Dark current	10	nA
	Thermal noise	10^−25^	W/Hz
	Temperature	300	K
	Shot noise	Yes	
	Width	10	µm
	Low pass filter cut-off frequency	0.75 × Bit rate	Hz
1:4 Power combiner/splitter	Insertion loss	2	dB

**Table 2:** Maximum achievable FSO range under different conditions in the proposed system @ 10^−9^ BER.

Weather	LG[0,0]	LG[0,10]	LG[0,20]	LG[0,30]
Clear air	1900 m	1600 m	1550 m	1200 m
Drizzle haze	1800 m	1510 m	1500 m	1100 m
Moderate haze	1550 m	1500 m	1500 m	1000 m

**Table 3:** Maximum temperature attained under different conditions in the proposed system @ 10^−9^ BER.

Weather	LG[0,0]	LG[0,10]	LG[0,20]	LG[0,30]
Clear air	100^0^	90^0^	90^0^	20^0^
Drizzle haze	80^0^	90^0^	80^0^	<20^0^
Moderate haze	110^0^	120^0^	110^0^	<20^0^

**Table 4:** Relationship between different attenuation parameters for gamma–gamma channel model.

Turbulence	$C_{n}^{2\;}$(m^−2/3^)	$k_{0}^{2}$	m	n	SI	SR
Weak	10^−17^	0.5	10	9	0.2	1
Medium	10^−14^	1.1	4.2	2.2	0.80	0.5
Strong	10^−12^	1.5	2.14	1.97	1.21	0

**Table 5:** Achieved Log(BER) values for varied index modulation under different models and turbulence effects at 1000 m range at 160 Gbps.

Turbulence	LG[0,0]		LG[0,10]		LG[0,20]		LG[0,30]	
	GG	LN	GG	LN	GG	LN	GG	LN
Weak	−21.99 to −3.88	−21.56 4.59	−21.61 to −4.6	−21.84 to -4.53	−21.6 to −4.53	−22.78 to −4.1	−22.53 to −3.85	−22 to −3.49
Medium	−16.16 to −3.51	−19.74 −4.35	−13.83 to −3.65	−19.4 to -4.23	−13.52 to −3.2	−19.75 to −4.25	−13.21 to −3.78	−19.75 to −4.04
Strong	−3.28 to −2.34	−3.9 −1.87	−4.35 to −2.14	−3.44 to −2.19	−3.78 to −2.26	−3.38 to −2.56	−4.11 to −2.33	−4.01 to −2.15

**Table 6:** Comparison analysis w.r.t. prior works.

Reference	Maximum Range	No. of Channels	Data Rate/ Channel (Gbps)	Mode(s)	Sensor	Operating Wavelength(s)/Frequency(s)	Weather Conditions	Turbulence	FSO Channel Model	Complexity and Cost
[9]	1.5 km (FSO)	1	120 Gbps	OAM	-	1550 nm	Low and heavy dust	Strong	Gamma- Gamma	High
[26]	11.6 km (SMF), 6 m (FSO)	-	20 Gbps	-	-	1609.98– 1610.84 nm	Fog, smoke	-	-	High
[10]	-	8	2 Gbps	OAM	-	-	-	Weak and strong	-	High
[1]	100 m	1	10 Gbps	-	Photonic	1529–1568.2 nm	-	-	-	Moderate
[8]	2 m (FSO)	4	2.5 Gbps	-	FBG	1520–1560 nm	-	-	-	High
[3]	25 km (SMF) + 2 m (FSO)	-	-	-	FBG	1544–1548 nm	-	-	-	High
[2]	-	-	-	-	FBG	1544.59, 1545.52 and 1546.53 nm	-	-	-	-
[5]	2 km (FSO)	1	-	-	-	1550 nm	Air, rain, snow	-	Malaga	Moderate
This work	1.9 km (FSO)	16	10 Gbps	LG	FBG	193.1, 193.2, 193.3 and 193.4 THz	Air, drizzle and moderate haze	Weak-to- strong	Gamma- Gamma, Log- Normal	Low

**Table 7:** Comparison analysis w.r.t. prior works in terms of system reliability and cost-effectiveness.

Reference	Input Power	Modulation and Detection	Channel Capacity	Aperture (Tx/Rx)	Loss	OSNR	Divergence Angle	BER Limit	Modes
[9]	20 dBm	OFDM	120 Gbps	10/10 cm	Not considered	21.9 dB	0.02 mrad	10^−3^	LG{[0], [0,10]}
[26]	11 dBm	Quadrature Phase Shift Keying (QPSK)	20 Gbps	-	Free space path	-	-	10^−3^	-
[10]	3.7 dBm	QPSK	8×2 Gbps	-	Mode-dependent loss	-	-	10^−3^	OAM [l = +1 & l = −2]
[1]	2.1 dBm	OOK	10 Gbps	-	Insertion, attenuation, coupling	-	-	-	-
[8]	-	-	4 × 2.5 Gbps	-	-	-	-	10^−9^	-
[5]	20 dBm	OOK	-	0.05 m (Rx)	Attenuation, pointing error	-	-	-	-
This work	10 dBm	OOK/DD	16 × 10 Gbps	10/20 cm	Transceiver, Geometric, Additional and Free space path	113.39 dB	2 mrad	10^−9^	LG{[0], [0,10], [0,20] and [0,30]}

## Data Availability

Data are contained within the article.
